# COVID-19: years of life lost (YLL) and saved (YLS) as an expression of the role of vaccination

**DOI:** 10.1038/s41598-022-23023-0

**Published:** 2022-10-28

**Authors:** Klára Hulíková Tesárková, Dagmar Dzúrová

**Affiliations:** 1 Department of Demography and Geodemography, Faculty of Sciences, Charles University, Prague, Czechia; 2 Department of Social Geography and Regional Development, Faculty of Sciences, Charles University, Prague, Czechia

**Keywords:** Viral infection, Lifestyle modification

## Abstract

When evaluating vaccine efficacy, the conventional measures include reduction of risk of hospitalization and death. The number of patients dying with or without vaccination is often in the public spotlight. However, when evaluating public health interventions or the burden of disease, it is more illustrative to use mortality metrics taking into account also prematurity of the deaths, such as years of life lost (YLL) or years of life saved (YLS) thanks to the vaccination. We develop this approach for evaluation of the difference in YLL and YLS between COVID-19 victims with or without completed vaccination in the autumn pandemic wave (2021, October–December) in Czechia. For the analysis, individual data about all COVID-19 deaths in the country (N = 5797, during the studied period) was used. While 40.6% of the deaths are in cohorts with completed vaccination, this corresponds to 35.1% of years of life lost. The role of vaccination is expressed using YLS and hypothetical numbers of deaths. The registered number of deaths is approximately 3.5 times lower than it would be expected without vaccination. The results illustrate that vaccination is more effective in saving lives than suggested by simplistic comparisons.

## Introduction

During the pandemic years, 2020 and 2021, Czechia has been ranked in the number of deaths per capita as one of the worst countries in the World (the total number of COVID-19 victims in a country of 10.5 million inhabitants surpassed 41 thousand in September 2022). There were 39,248 coronavirus-related deaths registered in the country since the pandemic began until March 15, 2022. This is staggeringly high compared to, for instance, Austria, a border country of a similar population size, which had less than half of the number of coronavirus-related deaths (14,609; March 14, 2022)^1^. The burden of the epidemic is most often assessed by the number of deaths, but this is only an inaccurate rough indicator, as neither the age structure of the population nor the age of the victims is taken into account^2^.

Demography works with many indicators that eliminate the influence of age structures and thus enable better and more correct regional or international comparisons. When evaluating public health interventions or the burden of disease, it is often preferable to use mortality metrics taking into account also how premature the deaths are, or the age structure of the victims in general, such as years of life lost (YLL). YLL is a valid measure in demography, potentially used also for identifying and classifying the underlying causes of premature mortality^3–5^. The method was probably first used in the Global Burden of Disease Study^3^.

The YLL method has already been used as a metric to evaluate the effects of COVID-19 in several published articles^6–8^. Pifarré et al. compared the effects of COVID-19 using YLL in 81 countries, including Czechia^6^. The authors concluded that in highly developed countries, the impact of COVID-19 was 2–9 times higher than for common seasonal influenza (compared to the median influenza year in the same country). For Czechia, it was concluded that for COVID-19 it was up to 5 times higher in 2020 in terms of the number of YLL than the effects of the common seasonal flu.

These publications and many others address the estimation of YLL in the context of the ongoing COVID-19 pandemic, but only a few address the use of the YLL method to evaluate the effect of vaccination against COVID-19^9^.

Misinformation and hesitancy in vaccines, potentially leading to refusal or delayed acceptance of COVID-19 vaccines, are considered a key factor in the high number of pandemic victims. When evaluating vaccine efficacy, the conventionally-evaluated outcomes include a reduction in the risk of hospitalization and death. The number of patients dying with or without completed vaccination is often also in the public spotlight. In this context, it is worth mentioning a unique study by Joshua Goldstein et al.^9^. Their results support the preference for vaccination among the oldest ages. The authors show that vaccinating the most vulnerable people will provide the highest protection against deaths, both in terms of the number of deaths and in terms of YLL, but also in terms of the number of years of life saved (YLS). At the time of preparation of the cited study^9^ the authors had to use the assumption of vaccination coverage and its efficiency for estimation of the overall supposed effects. At present, in developed countries, vaccines are available to everyone, regardless of age (except for children under 5 years of age). That means, that nowadays it is not necessary to base the study on many assumptions. Rather, it is possible to use the empirical data for estimation of the effects of vaccination (as in the analytical part of this paper). Still, however, the number of YLL or YLS could be used for its clearness and simplicity.

Because most people dying of COVID-19 are elderly, opinions resonate that YLL and YLS due to COVID-19 disease are low and that the young population is spared the serious consequences of the disease. For potential evaluation of such assumptions, younger ages are also included in the analysis below.

The efficacy of COVID-19 vaccines can in principle be assessed in terms of both YLL in the population without complete vaccination and YLS in the population with complete vaccination. We can set a more common question, how many years of life have been lost because of COVID-19 in the population with and without complete vaccination, as well as a less common question, how many years of life were saved among those who have complete vaccination against COVID-19. The measure of YLS through COVID-19 vaccination is used only sporadically, however, its construction is very straightforward as an alternative to YLL^9^.

We aim to estimate YLL and YLS in connection with COVID-19 using data on completed vaccination and individual deaths related to COVID-19 (See the Data and Methods section for proper definitions of terms and variables). This study quantifies the years of life lost and saved associated with completed vaccination in the period until the peak of the fifth Czech epidemic wave with dominantly only one variant—the Delta variant (October–December 2021)^10^. To our knowledge, this is a unique study on the effectiveness of vaccination using the method of estimating the YLL and YLS for the period of the Delta variant.

## Data and methods

For the purpose of the study, we used the continuously collected and published data related to COVID-19 pandemic in Czechia^11^. In the database, deaths related to COVID-19 are defined as the deaths of “individuals positively tested for SARS-CoV-2 (by PCR) regardless of the reasons for their deaths, and regardless of whether they died in a hospital or outside hospital care”^12^. Clearly, for not all of the registered deaths, the COVID-19 was the underlying cause of death, however, the official statistics based on the underlying causes are published with a much higher time delay and the numbers are not distinguished according to the vaccination status. For this reason, we used the continuously registered data where the deaths could be taken directly or indirectly related to the disease. These data are also included in international databases and correspond to the international standards of data evidence^12^.

The initial dataset used for analysis included individual death records related to the COVID-19 disease in the period of three months from October 1 to December 31, 2021 (N = 5797) by the time given by the availability of data at the time of analysis (March 2022). Data were obtained from the Czech National Information System, which includes records of all individuals who tested positive for SARS-CoV-2^12,13^.

The studied period was chosen for three main reasons: (1) only one variant, that is, the Delta variant was the dominant strain of COVID-19^10^; (2) the possibility of completed vaccination for all persons over 12 years of age; (3) the vaccines administered were suitable for the Delta variant^14^.

The following characteristics were available for all death records: age, sex, and information on the course of vaccination (date of the first, second, and booster vaccines). We used only population and numbers of deaths at the age of at least 12, because younger children were not vaccinated at that time in Czechia. The dataset was divided into two groups of cases according to the completed vaccination as follows: (a) persons who died without previously completed vaccination and (b) persons with completed vaccination at the time of death. At least one dose for a single-dose type of vaccine, or at least two doses for a two-dose vaccine, is considered the complete vaccination. Deaths of persons with incomplete vaccination (only one dose for a two-dose vaccine) are included in the first group (“without previously completed vaccination”), they are not studied separately as there are low numbers of cases.

### Estimated years of life lost and saved

A measure of disease burden—expected years of life lost (YLL) is often used for comparative purposes. In the calculation, each death is weighted as a function of age at the time of death, reflecting the fact that deaths at young ages are related to a higher number of years of life lost (i.e. longer average lengths of potential remaining life lost) than deaths at an advanced age^4,5^. The potential remaining length of life was estimated using the age-specific life expectancy according to sex before the onset of the pandemic in 2019 (${e}_{x, 2019}$), as published by the Czech Statistical Office^15^.

Because several chronic diseases or health states such as obesity and diabetes mellitus are considered as factors increasing the risks of severe outcomes of COVID-19, it could be speculated that people who have died from the COVID-19 disease have usually been comorbid, with more serious diseases that are in themselves associated with reduced life expectancy. In that case, it seems rather inappropriate to use the average population life expectancy as the potential length of the remaining life of the population deceased in relation to COVID-19. Their potential remaining length of life could be expected to be on average shorter, however, the shortening could be only supposed or roughly estimated, as there are no data available for its calculation.

That is why the potential years of life lost or years of life saved are estimated using three scenarios based on different assumptions of the potentially remaining length of life. The potential remaining length of life in all of them is based on official life tables for the year 2019^15^.

In the first one, the baseline scenario, the life expectancy (${e}_{x, 2019,}$) is used as an estimation of the potential remaining years of life for all of the deceased persons in relation to COVID-19. As mentioned above, this assumption seems to be rather overestimated in relation to the potential remaining length of life.

In the second scenario, we used the 70th percentile of remaining expected years of life for each sex and age from the official life tables for the year 2019^15^ (i.e. we used the potential remaining length of life as the number of years of life within which the first 30% of the population aged *x* die according to the distribution of the survival function, ${l}_{x}$, in the life tables, ${e}_{x, 2019, P70}$). In general, we used the calculation as

1 $${e}_{x, 2019, P}={x}_{P}+\frac{{l}_{{x}_{P}}-P\times {l}_{x}}{{l}_{{x}_{P}}-{l}_{{x}_{P}+1}}$$

where *P* = *0.7* for the 70th percentile, ${l}_{x}$ is the survival function of the life tables $, {x}_{P}$ is the highest age where ${l}_{x}$ is higher than $P\times {l}_{x}$.

In the third scenario, the 90th percentile was used, where the potential remaining length of life at age *x* was set as the value corresponding to the death of the first 10% of the population at that age (based again on life tables 2019, ${e}_{x, 2019, P90}$ calculated as in Eq. (1) where *P* = 0.9).

Except for the baseline scenario, the above-mentioned third scenario (based on ${e}_{x, 2019, P90}$) could be taken as rather a pessimistic one according to assumptions of the potential remaining length of life, and the second scenario (based on ${e}_{x, 2019, P70}$) may provide the closest real potential. When dealing with the scenarios, it is important to keep in mind that the main aim is to compare the overall trends or crucial differences given by the initial assumptions of the scenarios, not to discuss the detailed resulting values based on estimations and assumptions.

**Figure 1:**
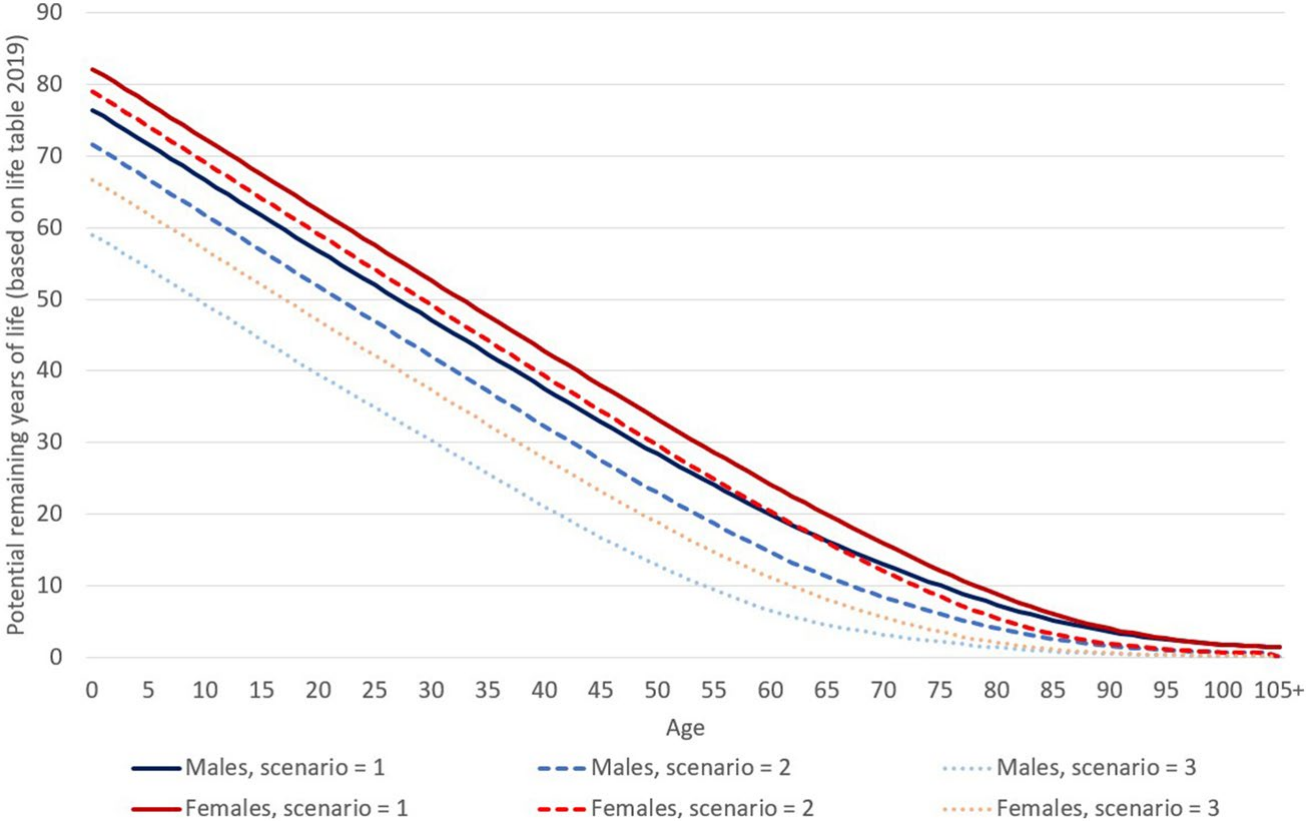
Potential years of remaining life (Eq. 1) according to age used in the three scenarios of the analysis for males and females based on the life tables for the year 2019. Source: author’s calculation according to Eq. (1)^15^.

In the equations, the number of deaths at age *x* is marked as ${D}_{ x}$. The calculation was processed separately for males and females, and for detail of individual ages, values of YLL were then aggregated according to below-defined age groups.

2 $${YLL}=\sum_{x}{YLL}_{ x}=\sum_{x}{D}_{ x} *{e}_{x, 2019, scenario=i}$$

where ${e}_{x, 2019, scenario=i}$ is equal to ${e}_{x, 2019}$ for the baseline scenario, it is equal to ${e}_{x, 2019, P70}$ for *i* = 2 or ${e}_{x, 2019, P90}$ for *i* = 3. Values of life expectancy (${e}_{x, 2019}$) for males and females, as well as ${e}_{x, 2019, P70}$ and ${e}_{x, 2019, P90}$ are presented in Fig. 1. On average, a man dying at the age of 65 loses 16.3 potential years of life in scenario 1 (corresponds to the life expectancy at age 65 for males in 2019), 11.3 years in scenario 2, and 4.6 years in scenario 3. For females, those three values would be 19.9 in scenario 1, 16.1 in scenario 2, and 8.1 years in scenario 3. Based on Eq. (2), the potential remaining years of life represents a weight of the number of deaths at each age.

Years of life saved (YLS) through COVID-19 vaccination are proposed as a (more optimistic and, therefore, potentially more publicly acceptable) measure of the effect of vaccination on COVID-19-related mortality. Since this is the estimated effect of vaccination, the value of YLS is calculated only for the sub-population with completed vaccination. In this paper, it was estimated as a difference between the hypothetical years of life lost (${YLL}_{HYP}$) in the sub-population with completed vaccination and years of life lost based on registered deaths related to COVID-19 in the population with completed vaccination (${YLL}_{vac}$) (Eq. 3).

3 $$YLS={YLL}_{HYP}-{YLL}_{vac}$$

The hypothetical years of life lost (${YLL}_{HYP}$) are calculated only for the population with completed vaccination, and it is based on the assumption that the risk of COVID-19-related death in the population with completed vaccination was equal to the risk of COVID-19-related death in the population without completed vaccination (Eq. 4).

The first step in the calculation of the hypothetical years of life lost (${YLL}_{HYP}$) is the estimation of the hypothetical number of deaths at age *x* (${D}_{HYP, x}$) under the assumption that the risk of death in the population with completed vaccination was equal to the risk of death in the population without completed vaccination. It was estimated using the population size with completed vaccination according to age and sex (${P}_{vac, x}$) and age-specific quotients of lethality (risk of death) from COVID-19 in the population without completed vaccination (${lq}_{unvac, x}$):

4 $${D}_{HYP, x}= {P}_{vac, x}* {lq}_{unvac, x}={P}_{vac, x}*\frac{{D}_{unvac, x}}{{P}_{unvac, x}}$$

where ${P}_{vac, x}$ and ${P}_{unvac, x}$ are the estimated population sizes according to age (*x*) at the beginning of the studied period (October 1, 2021) with completed vaccination (*vac*) and without completed vaccination (*unvac*) using the numbers of fully vaccinated persons based on the official evidence^11^. Where ${D}_{unvac, x}$ is the registered number of deaths at age *x* related to COVID-19 in the population without completed vaccination, the age-specific quotient of lethality (${lq}_{unvac, x}$) represents the risk of an person without completed vaccination dying from COVID-19 during the studied period.

If the risk of death of the population with completed vaccination as well as the population without completed vaccination was the same (assumption of the null effect of completed vaccination), the number of deaths among the population with completed vaccination would equal to ${D}_{HYP, x}$. Using the average number of years of life lost per death from the population without completed vaccination ($\frac{{YLL}_{unvax, x}}{{D}_{unvax, x}}$) the hypothetical years of life lost is calculated as (second step of the calculation, Eq. 5):

5 $${YLL}_{HYP}=\sum_{x}{D}_{HYP, x}*\frac{{YLL}_{unvax, x}}{{D}_{unvax, x}}$$

In the calculations of years of life saved (YLS), we used the age groups (12–44, 45–64, 65–84, 85 +) instead of individual ages, and all the calculations were also processed separately for males and females. Also, other results are presented for the defined age groups. That is, the age group labeled as 85 + represents the oldest population aged 85 and more years, most often affected by chronic diseases, etc. Younger seniors are represented by the age group 65–84 years. The younger ages are further divided into the age 12–44 years, where only a marginal part of deaths occurs, and the age group 45–64 years which could still be considered as a relatively young age group (in the age of economic activity). However, this age group had already been significantly affected by the pandemic (see below, Table 1).

**Table 1:** Registered numbers of deaths within the population with completed vaccination and without completed vaccination by males, female and, both sexes during the period from October 1 to December 31, 2021.

	Males		Females		Both sexes	
	Without completed vaccination	With completed vaccination	Without completed vaccination	With completed vaccination	Without completed vaccination	With completed vaccination
**Absolute numbers of registered deaths related to COVID-19**						
Age 12–44	37	6	16	2	53	8
Age 45–64	368	87	181	49	549	136
Age 65–84	1076	982	934	578	2010	1560
Age 85 +	277	323	552	329	829	652
Total	1758	1398	1683	958	3441	2356
**Relative age structure of registered deaths related to COVID-19 (in %)**						
Age 12–44	2.1	0.4	1.0	0.2	1.5	0.3
Age 45–64	20.9	6.2	10.8	5.1	16.0	5.8
Age 65–84	61.2	70.2	55.5	60.3	58.4	66.2
Age 85 +	15.8	23.1	32.8	34.3	24.1	27.7
Total	100.0	100.0	100.0	100.0	100.0	100.0

Numbers are absolute numbers, and the relative structure is expressed as (%).

Source: author’s calculation^11^.

All methods were carried out in accordance with relevant guidelines and regulations; no experiments on humans were done, and no human tissue samples or data were used**.**

### Ethics declarations

Data are routinely collected in compliance with Czech legal regulations (Act on the Protection of Public Health). To use anonymized, retrospective data from this database there is no need for ethical approval.

## Results

Tables 1 and 2 summarise the total number of registered deaths related to the COVID-19 disease by age groups and vaccination status (${D}_{vac}$ and ${D}_{unvac}$), and also the estimation of the hypothetical number of deaths ${D}_{HYP}$ (Table 2). In the analyzed period from October 1 to December 31, 2021, there were reported 5797 deaths in Czechia, of which 3441 deaths were in the population without complete vaccination (59.4% of the total number). In terms of age, the highest number of victims was aged 65–84 (3570), followed by the oldest age group 85 and over (1481). There were 61 deaths between the ages of 12 and 44. In terms of vaccination, the relative distribution was different. The highest proportion of victims without completed vaccination was in the lowest age category (86.9% of the registered 61 deaths at age 12–44). In the age group of 45–64 years, 549 people (80.1%) died without completed vaccination, and 136 people (19.9%) died with completed vaccination (Table 1).

**Table 2:** Estimated population sizes at the beginning of the studied period (October 1, 2021) with and without completed vaccination, hypothetical numbers of deaths (population with complete vaccination) and estimated saved numbers of death (population with complete vaccination).

	Males		Females		Both sexes	
	Without completed vaccination	With completed vaccination	Without completed vaccination	With completed vaccination	Without completed vaccination	With completed vaccination
**Estimated population sizes at the beginning of the studied period (October 1, 2021) with and without completed vaccination**						
Age 12–44	1,125,380	1,093,933	1,051,331	1,036,839	2,176,711	2,130,772
Age 45–64	440,973	1,005,131	415,616	1,007,147	856,589	2,012,278
Age 65–84	120,549	726,934	172,025	935,425	292,574	1,662,359
Age 85 +	7462	54,506	26,173	115,248	33,635	169,754
Total	1,694,364	2,880,504	1,665,145	3,094,659	3,359,509	5,975,163

	Males	Females	Both sexes			
**Hypothetical number of deaths in the population with completed vaccination under the assumption that the risk of death of the population with completed vaccination was equal to the risk of death of the population without completed vaccination**						
Age 12–44	36	16	52			
Age 45–64	839	439	1277			
Age 65–84	6488	5079	11,567			
Age 85 +	2023	2431	4454			
Total	9387	7964	17,350			
**Estimated saved (avoided) number of deaths in the population with completed vaccination (difference between the hypothetical and registered number of deaths)**						
Age 12–44	30	14	44			
Age 45–64	752	390	1 141			
Age 65–84	5506	4501	10,007			
Age 85 +	1700	2102	3802			
Total	7989	7006	14,994			

Source: author’s calculation^11,15^.

However, absolute numbers of deaths in individual categories are influenced by the structure of the exposed population according to vaccination coverage and age (Table 2). By October 1, 2021, approximately 64% of the population aged 12 and older had completed vaccination. The highest coverage by completed vaccination was at the highest age groups—around 83.5% at age 85 + and around 85.0% at age 65–84. Only 70.1% of the population aged 45–64 had completed vaccination, and 49.5% were in the youngest age group (12–44 years).

**Figure 2:**
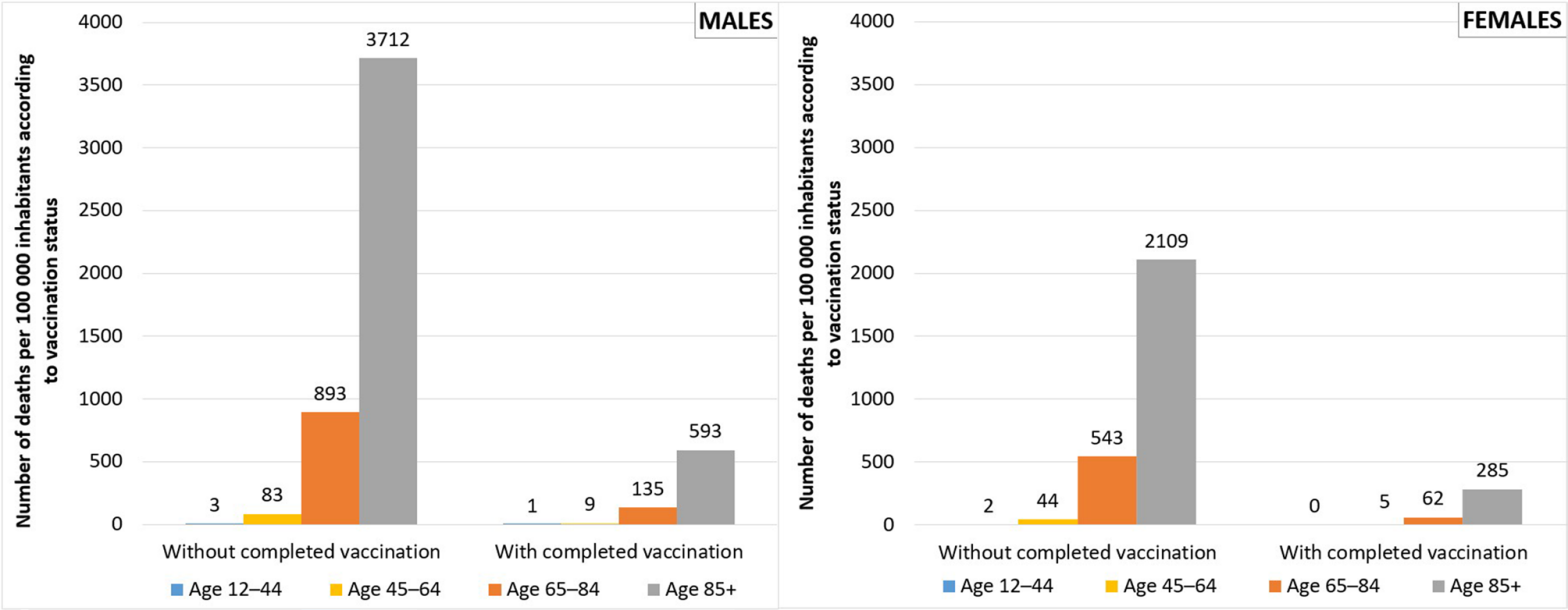
Number of deaths per 100,000 inhabitants according to vaccination—population with and without completed vaccination, males (left), females (right). Source: author’s calculation^11,15^.

Table 2 gives the estimates of hypothetical deaths in the population with a completed vaccination (${D}_{HYP}$) for each age group and sex, assuming that the risk of death of the population with completed vaccination equals the risk of death of the population without completed vaccination. However, the population with completed vaccination had considerably lower rates of COVID-19 mortality (on average 7.5 times less, see below and Fig. 2). For a comparison, the age-specific quotients of lethality (risk of death) from COVID-19 in the population without completed vaccination (${lq}_{unvac, x}$) and with completed vaccination (${lq}_{vac, x}$) were calculated. These measures are presented in Fig. 2 as a number of registered deaths per 100,000 inhabitants according to vaccination status for males and females, in particular, defined by age groups.

In Fig. 2, it is clear that the risk of death related to COVID-19 significantly increases with age, however, the pace is higher in the population without completed vaccination. If we do not consider the youngest age group (12–44) where the numbers of death are small, the risk of death is 6.4-times higher for the population without completed vaccination aged 85 and older as compared to the population with completed vaccination for the same age group. In the age group 65–84 years, the risk is 7.3-times higher, and in the age group 45–64 it is even 9.5-times higher for the sub-population without completed vaccination (Fig. 2).

### Years of life lost (YLL)

**Figure 3:**
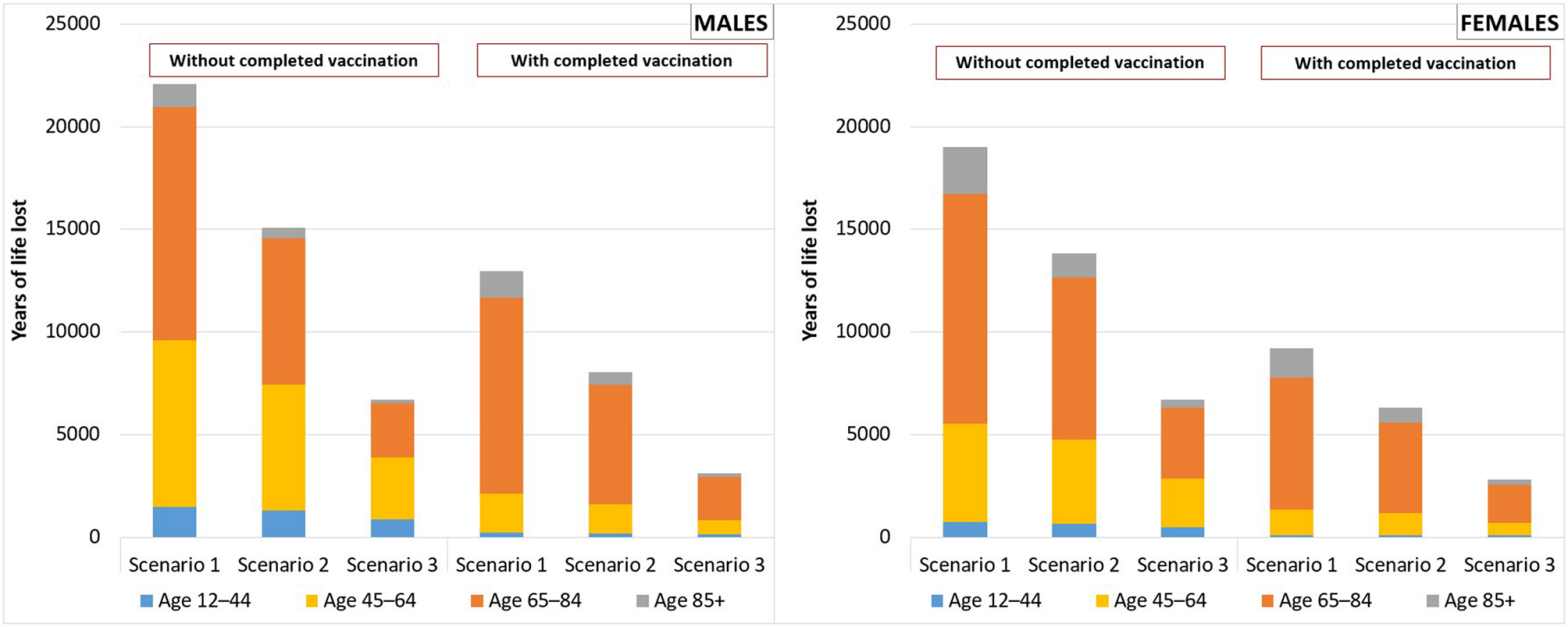
Years of life lost (YLL) by males, females, population with or without completed vaccination, and Scenarios 1–3 during the period of October 1 to December 31, 2021, Czechia. Source: author’s calculation^11,15^.

The total number of deaths (5797) led to more than 63 thousand years of life lost in the baseline scenario 1 (where the lost potential length of life equals to life expectancy in 2019) while the total number of years of life lost reached more than 43 thousand in alternative scenario 2 and more than 19 thousand in alternative scenario 3. In terms of the number of YLL, Fig. 3 shows an even more pronounced effect of vaccination, i.e. a significantly lower number of years of life lost in the sub-population with completed vaccination. Two-thirds (64.9% in scenario 1, 66.8% in scenario 2, and 69.3% in scenario 3) of the years of life lost belong to the victims without completed vaccination. What might even be more important, a significantly higher proportion of the years of life lost among the population without completed vaccination is caused by the deaths at the age of economic activity (below 65 years). Among males, it is around 50% of YLL, and among women it is around one-third of YLL. Considering the population with completed vaccination, the proportion of YLL at the age below 65 is around 20% (for both sexes). More detailed results are included in the Supplementary information S1.

### Years of life saved (YLS)

Table 3 provides an overview of the three calculated indicators for the population with completed vaccination, the hypothetical number of years of life lost (${YLL}_{HYP}$), registered years of life lost (${YLL}_{vac}$), and years of life saved (${YLS}$) by age and sex. The measure of years of life saved (YLS) enables the evaluation of the effect of vaccination in a less usual way.

**Table 3:** The hypothetical years of life lost (${YLL}_{HYP}$), years of life lost (${YLL}_{vac}$), and years of life saved through COVID-19 vaccination ($YLS$) in the population with completed vaccination during the period of October 1 to December 31, 2021, Czechia, Scenarios 1–3.

	Males	Females	Both sexes	Males	Females	Both sexes	Males	Females	Both sexes	Males	Females	Both sexes
	*YLL*_*HYP*_			*YLL*_*vac*_			*YLS*			Proportion of YLS from hypothetical YLL (in %)		
**Scenario 1**												
Age 12–44	1428.7	704.1	2132.7	224.7	102.3	327.0	1204.0	601.7	1805.7	84.3	85.5	84.7
Age 45–64	18,493.4	11,620.6	30,114.0	1871.7	1240.3	3112.0	16,621.7	10,380.3	27,002.0	89.9	89.3	89.7
Age 65–84	68,557.0	60,881.4	129,438.4	9581.6	6423.2	16,004.9	58,975.3	54,458.2	113,433.5	86.0	89.4	87.6
Age 85 +	8076.1	10,183.4	18,259.4	1295.7	1434.9	2730.6	6780.4	8748.5	15,528.8	84.0	85.9	85.0
Total	96,555.1	83,389.5	179,944.6	12,973.7	9200.8	22,174.5	83,581.4	74,188.7	157,770.1	86.6	89.0	87.7
**Scenario 2**												
Age 12–44	1240.5	648.7	1889.2	193.1	95.5	288.5	1047.4	553.2	1600.6	84.4	85.3	84.7
Age 45–64	14,072.0	9954.8	24,026.8	1414.5	1053.4	2467.9	12,657.5	8901.4	21,559.0	89.9	89.4	89.7
Age 65–84	42,755.4	43,005.5	85,760.9	5839.1	4429.3	10,268.4	36,916.3	38,576.3	75,492.6	86.3	89.7	88.0
Age 85 +	3773.6	5036.6	8810.2	605.8	718.1	1324.0	3167.8	4318.4	7486.2	83.9	85.7	85.0
Total	61,841.5	58,645.6	120,487.1	8052.5	6296.3	14,348.8	53,789.0	52,349.3	106,138.3	87.0	89.3	88.1
**Scenario 3**												
Age 12–44	832.7	465.8	1298.5	125.9	71.9	197.8	706.8	393.9	1100.6	84.9	84.6	84.8
Age 45–64	6904.1	5775.2	12,679.3	683.8	598.6	1282.4	6220.3	5176.6	11,396.9	90.1	89.6	89.9
Age 65–84	15,816.4	18,718.2	34,534.6	2122.8	1879.5	4002.4	13,693.5	16,838.7	30,532.2	86.6	90.0	88.4
Age 85 +	1215.7	1662.4	2878.1	195.2	238.0	433.1	1020.5	1424.5	2445.0	83.9	85.7	85.0
Total	24,768.8	26,621.6	51,390.4	3127.7	2788.0	5915.7	21,641.0	23,833.6	45,474.6	87.4	89.5	88.5

Source: author’s calculation^11,15^.

In all three scenarios, the highest number of YLS could be observed in the age group 65–84 years. However, in relative presentation, the proportion of YLS from the hypothetical YLL, i.e. the effect of vaccination, is similar across all the age groups and all three scenarios—it is around 85–90% (Table 3).

Under the assumption of no effect of vaccination, the numbers of deaths related to COVID-19 in the population with completed vaccination would have been almost 15 thousand higher during the studied period. That is, without vaccination against COVID-19, the number of deaths related to COVID-19 would probably have been 3.5 times higher in the analyzed period (Table 2). Through vaccination, in total around 88% of the hypothetical number of YLL was prevented among the inhabitants with completed vaccination. An equally important result of the study is the outcome that the proportions of years of life saved by vaccination from the overall hypothetical years of life lost are almost identical for all age groups (from 85 to 89%, Table 3). The observed results are also robust across all the scenarios. The conclusions do not depend on initial assumption related to potential remaining length of life.

## Limitations

As with any other studies, this study has to deal with some limitations. From the methodological point of view, it needs be mentioned that the evidence of deaths related to COVID-19 cannot be 100% complete because some deaths are not defined precisely according to the cause of death. Some cases of COVID-19 positivity were not revealed because of limited testing, for example. In this study, we do not consider the period from vaccination completion which may play a significant role in the vaccine efficiency^16^ as well as the lag between vaccination and its protective effect^17^. This remains the potential object in another study focused more on the time dimension and duration of the effects of vaccines. Also, we have no information about the health status or comorbidities of the victims or of the vaccinated and unvaccinated population in general.

## Conclusion

The COVID-19 pandemic emphasized the need of interdisciplinary approach. The role of demography is irreplaceable in the case of evaluation of its consequences for the population and the effectiveness of applied measures above all. The study has developed a method usable in this evaluation and contributes to the topic of vaccine effectiveness using demographic and mathematical methods. Metrics such as YLL should be considered when evaluating the impacts of various population-wide interventions. Assessing years of life lost is a good indicator of the effects of a pandemic, as it provides a much more relevant view than the crude mortality rate (numbers of deaths per population size) often used in practice. In this study, the measure of YLL was used not only for illustration of the outcome of the pandemics but above all for the evaluation of the effect of completed vaccination. It provides clear evidence of the benefits of COVID-19 vaccination, and using the YLS illustrates the advantage of the population with complete vaccination as compared to the population without it.

From a public health perspective, not only are the years of life lost assessing how much life years have been shortened for populations affected by the COVID-19 disease, but equally important are the years of lives saved by interventions—COVID-19 vaccination in case of this study. This study quantifies the years of life lost and saved associated with completed vaccination in the period until the peak of the fifth Czech pandemic wave (October–December 2021), when 5797 people died from the COVID-19 disease.

This result illustrates that vaccination is even more effective in saving lives than suggested by straightforward and often simplified comparisons. Moreover, in the case of Czechia, among the population with completed vaccination, almost 15 thousand COVID-19-related deaths were potentially avoided. Vaccination helped to reduce the YLL among the fully vaccinated by around 88% during the studied period and the registered number of deaths is approximately 3.5 lower than it would be expected without vaccination.

This study demonstrates that COVID-19 vaccination saves lives and saves years of potential future lives.

## Supplementary Information

Supplementary Information.

## Data Availability

Data used in this study for the analyses are not publicly available. De-identified individual-level data are available to the scientific community (authorized access only after registration at www.uzis.cz/index-en.php). All the following calculations were prepared in MS Excel using the equations described in the text of the paper. The datasets analyzed in the study are available in the repository of the Ministry of Health of the Czech Republic (https://onemocneni-aktualne.mzcr.cz/covid-19), and of the Czech Statistical Office (Complete life tables for the Czech Republic for 2019, https://www.czso.cz/csu/czso/life-tables-for-the-czech-republic-cohesion-regions-and-regions-2018-2019). Ethical approval was not required for this secondary analysis of data publicly available the Czech National Information System.
